# Trends in survival for teenagers and young adults with cancer in the UK 1992–2006

**DOI:** 10.1016/j.ejca.2015.06.112

**Published:** 2015-09

**Authors:** C. O’Hara, A. Moran, J.S. Whelan, R.E. Hough, C.A. Stiller, M.C.G. Stevens, D.P. Stark, R.G. Feltbower, M.G. McCabe

**Affiliations:** a Clinical Outcomes Unit, The Christie NHS Foundation Trust, Wilmslow Road, Manchester M20 4BX, UK; b Public Health England, The Palatine Centre, 63-65 Palatine Road, Withington, Manchester M20 3LJ, UK; c NIHR University College London Hospitals Biomedical Research Centre, UCL Hospitals NHS Foundation Trust, 250 Euston Road, London NW1 2PG, UK; d Public Health England, 4150 Chancellor Court, Oxford Business Park South, Oxford OX4 2GX, UK; e School of Clinical Sciences, University of Bristol, Level 6, UHB Education Centre, Upper Maudlin Street, Bristol BS2 8AE, UK; f Leeds Institute of Cancer and Pathology, Cancer Genetics Building, St James’s University Hospital, Beckett Street, Leeds, LS9 7TF, UK; g Division of Epidemiology and Biostatistics, School of Medicine, University of Leeds, Worsley Building, Clarendon Way, LS2 9JT, UK; h Centre for Paediatric, Teenage and Young Adult Cancer, Institute of Cancer Sciences, University of Manchester, Young Oncology Unit, The Christie NHS Foundation Trust, Wilmslow Road, Manchester M20 4BX, UK

**Keywords:** TYA, Teenage, Adolescent, Cancer, Survival, Trends, UK, Outcomes

## Abstract

**Background:**

Although relatively rare, cancer in teenagers and young adults (TYA) is the most common disease-related cause of death and makes a major contribution to years of life lost in this age group. There is a growing awareness of the distinctive needs of this age group and drive for greater understanding of how outcomes can be improved. We present here the latest TYA survival trends data for the United Kingdom (UK).

**Methods:**

Using national cancer registry data, we calculated five-year relative survival for all 15–24 year olds diagnosed with cancer or a borderline/benign CNS tumour in the UK during the periods 1992–1996, 1997–2001 and 2002–2006. We analysed trends in survival for all cancers combined and for eighteen specified groups that together represent the majority of TYA cancers. We compared our data with published data for Europe, North America and Australia.

**Results:**

Five-year survival for all cancers combined increased from 75.5% in 1992–1996 to 82.2% in 2002–2006 (*P* < 0.001). Statistically significant improvements were seen for all disease groups except osteosarcoma, rhabdomyosarcoma, non-gonadal and ovarian germ cell tumours and ovarian and thyroid carcinomas. During the earliest time period, females had significantly better survival than males for five of the twelve non-gender-specific disease groups. By the latest period, only melanomas and non-rhabdomyosarcoma soft tissue sarcomas had differential survival by gender. Survival in the UK for the most recent period was generally similar to other comparable countries.

**Conclusion:**

Five-year survival has improved considerably in the UK for most cancer types. For some disease groups, there has been little progress, either because survival already approaches 100% (e.g. thyroid carcinomas) or, more worryingly for some cancers with poor outcomes, because they remain resistant to existing therapy (e.g. rhabdomyosarcoma). In addition, for a number of specific cancer types and for cancer as a whole males continue to have worse outcomes than females.

## 1 Introduction

Teenage and young adult (TYA) patients represent an important and distinct group within the overall population of cancer patients. Cancer is the most common cause of non-accidental death in 13–29 year olds [1] and makes a major contribution to years of life lost in this age group, with substantial economic consequences. There is now growing clinical awareness of the distinctive care needs of this age group and a concern that improvements in survival rates appear to be slower for TYA than for children and adults aged 45 years and older [2]. Moreover, low participation in clinical trials is impeding the development of optimal therapies for this age group [3,4].

Successive Eurocare projects have provided data on cancer survival in Europe by sub-region, including the United Kingdom (UK) and its constituent countries [5]. However, the latest TYA data reported are for patients diagnosed up to 2002. Survival data for England were also published in 2008 [6], for patients diagnosed up to 2001. More contemporary survival rates for adolescent and young adult cancer patients have been reported for several single European countries (e.g. [7–9] and for the United States (USA) [10]).

Since clinical trial enrolment is not the norm in this age group, and for many patients no suitable trials are available, there is a demand for clinical outcomes to be monitored using contemporary national data. We present here the most recent TYA survival data with mature follow up for the whole of the UK, for eighteen types of cancer that together represent 92% of all cancer diagnoses in the 15–24 year age group. We also compare our survival rates and trends for the UK with the most recent published data from other countries.

In the last decade, Clinical Service Guidance (CSG) for services for children, teenagers and young adults with cancer have been issued for England and Wales [11]. Specialised TYA Principal Treatment Centres have been established to oversee the care of TYA cancer patients, alongside more local TYA Designated Units for older TYA patients who choose to be treated closer to home. Our data largely pre-date the implementation of these guidelines. Therefore, in addition to showing the rate of improvement prior to the implementation of the CSG, we provide an important baseline estimate from which the effects of the implementation of national guidance can be evaluated and future international comparisons made.

## 2 Methods

Information on 25,658 patients aged 15–24 years diagnosed between 1992 and 2006 in the United Kingdom with a malignancy or a borderline/benign CNS tumour (such as pilocytic astrocytoma or schwannoma) was extracted from the UK National Cancer Data Repository (www.ncin.org.uk), an amalgamated dataset from the national cancer registries of England, Wales, Scotland and Northern Ireland. All non-melanoma skin carcinomas were excluded and only the first cancer for each patient was retained. Cancers registered only from death certificate data (DCOs) were discounted as were diagnoses where the date of death equalled the date of diagnosis in order to account for any inconsistencies between recording of DCOs between regions; 2% of cases for period 1 and 1% of cases for the two latter periods were excluded. Each case was censored for follow-up at 31st December 2011 or at earlier death (from any cause). Follow up is passive. Information on deaths is routinely received by UK registries from the relevant government department. This is added to the records held by the cancer registries. This system ensures complete follow up of cases with the exception of migration or rare losses.

Data for Northern Ireland (NI) are available for 1993 onwards only. Cases from NI represent less than 4% of the overall UK TYA cancer population. Based on data for 1997–2001 and 2002–2006, we estimated the absence of one year’s data for NI would have no significant impact on the overall UK survival rates and therefore decided against using any imputation methods and to include the full fifteen year period 1992–2006.

Cancer registrations in the UK are classified using either the WHO International Classification of Disease, Tenth Revision (ICD-10) or the WHO International Classification of Disease for Oncology (ICD-O) for site of tumour, along with ICD-O for tumour morphology. We used morphology codes converted to the second edition of ICD-O (ICD-O2) for consistency and grouped the diagnoses according to the standard classification for TYA cancers, combining registered site and morphology codes [12]. Soft tissue sarcomas were separated into rhabdomyosarcomas and non-rhabdomyosarcoma soft tissue sarcomas (NRSTS), since their management differs.

Five-year relative survival was estimated for all cancers combined and for each of the eighteen most common cancers for each five-year time period (1992–1996, 1997–2001 and 2002–2006). We analysed the population as a whole, and separately for males and females. Relative survival was calculated by the Ederer II method [13] using the strs command developed by the Dickman lab [14] in a Stata 12 environment. Differences in excess mortality at 5 years were tested for three time periods (1) 1992–1996, (2) 1997–2001 and (3) 2002–2006 using a multiple regression approach based on generalised linear models (GLM), assuming a Poisson distribution for the observed number of deaths [14], including age and period as covariates. We fitted the poisson regression model to the grouped data generated by the strs [14] command. Periods 2 and 3 were separately compared against period 1 as the baseline. Differences between males and females for each period were also tested separately using a GLM Poisson model including age and gender as main effects. Time- and gender-related differences were considered statistically significant at *P* < 0.05. All statistical analyses were conducted in Stata version 12.

Average annual percentage changes (AAPC) in five-year survival between 1992 and 2006 were calculated using the ‘Joinpoint’ software [15] with an overall significance level of alpha = 0.05, and a maximum of two joinpoints [16]. These data are presented for fifteen of the eighteen cancer types. Three cancer types were excluded from the analysis because the numbers of cases and deaths were too small to give meaningful results (ovarian germ cell tumours (GCTs) and non-gonadal GCTs) or because survival was consistently high throughout all study periods (thyroid carcinomas).

International comparisons were based on a literature search of MEDLINE databases covering the period 2005–2013. We selected publications of 5-year survival that

(a) were population-based studies using registry data,
(b) provided results for teenagers and young adults as a separate age category and for males and females combined, and
(c) covered a time period that overlapped with the most recent period (2002–06) of our study.

Comparisons were effected on a pragmatic basis whereby UK rates were considered to be similar to those of another country if the respective 95% confidence intervals overlapped. We made no attempt to make statistical inferences. Our observations should be considered in the context of the width of the confidence intervals and the relative population sizes. Because we were not able to directly compare 5-year survival between countries for identical time periods and were therefore not able to carry out formal statistical comparisons, we elected to include only the most recent UK survival data. More formal comparisons of survival across Europe have been published for earlier time periods [5].

## 3 Results

### 3.1 Study subjects

Table 1 shows the number of patients included in the study by type of cancer and by five-year period of diagnosis.

### 3.2 Trends in survival in the UK

Five-year relative survival rates are described in Table 2 for the three time periods for all cancers combined and for the eighteen most common cancers. For all cancers combined, survival increased significantly from 75.7% in 1992–1996 to 82.2% in 2002–2006 (*P* < 0.001) with significant improvements in both males and females.

### 3.3 Survival by cancer type

**Fig. 1:**
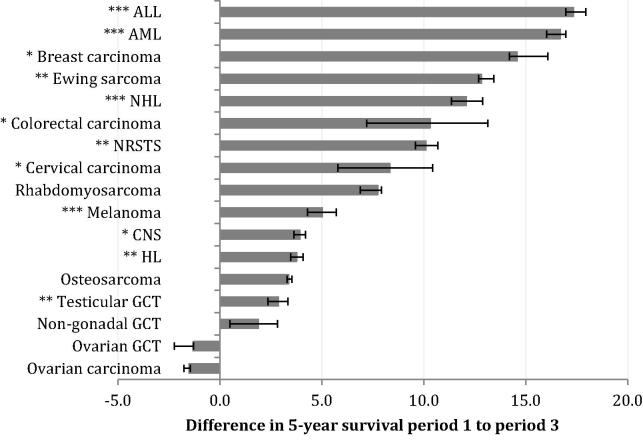
Differences in 5-year survival for all persons between 1992–1996 and 2002–2006. Significance is indicated as ^∗^*P* < 0.05, ^∗∗^*P* < 0.01, ^∗∗∗^*P* < 0.001. Error bars represent 95% confidence intervals. Thyroid carcinoma is excluded as baseline survival was already 100%. ALL, acute lymphoblastic leukaemia; AML, acute myeloid leukaemia; NHL, non-Hodgkin lymphoma; HL, Hodgkin lymphoma; GCT, germ cell tumour; CNS, central nervous system tumour; NRSTS, soft tissue sarcomas excluding rhabdomyosarcomas.

**Fig. 2:**
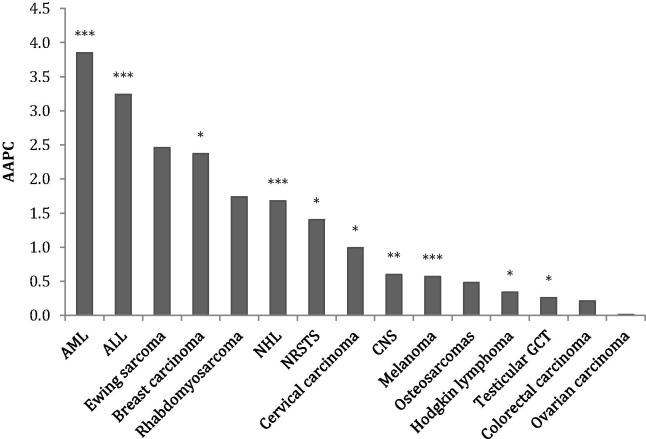
Annual average percentage change (AAPC) 1992–2006. Significance is indicated as ^*^P < 0.05, ^**^P < 0.01, ^***^P < 0.001. ALL, Acute lymphoblastic leukaemia; AML, acute myeloid leukaemia; NHL, non-Hodgkin lymphoma; HL, Hodgkin lymphoma; GCT, germ cell tumour; CNS, central nervous system tumour; NRSTS, soft tissue sarcomas excluding rhabdomyosarcomas.

Five-year survival varied markedly by cancer group. There were significant improvements in survival between 1992–1996 and 2002–2006 for twelve of the eighteen cancer types analysed (Fig. 1), including seven with improvements greater than 10%. Ten cancer types also had statistically significant changes in AAPC over time (Fig. 2). For Ewing sarcoma and colorectal carcinoma, although there was a significant improvement in survival between the earliest and latest time periods, the AAPC was not significant; survival improved significantly between periods 1 and 2 but not between periods 2 and 3.

**Fig. 3:**
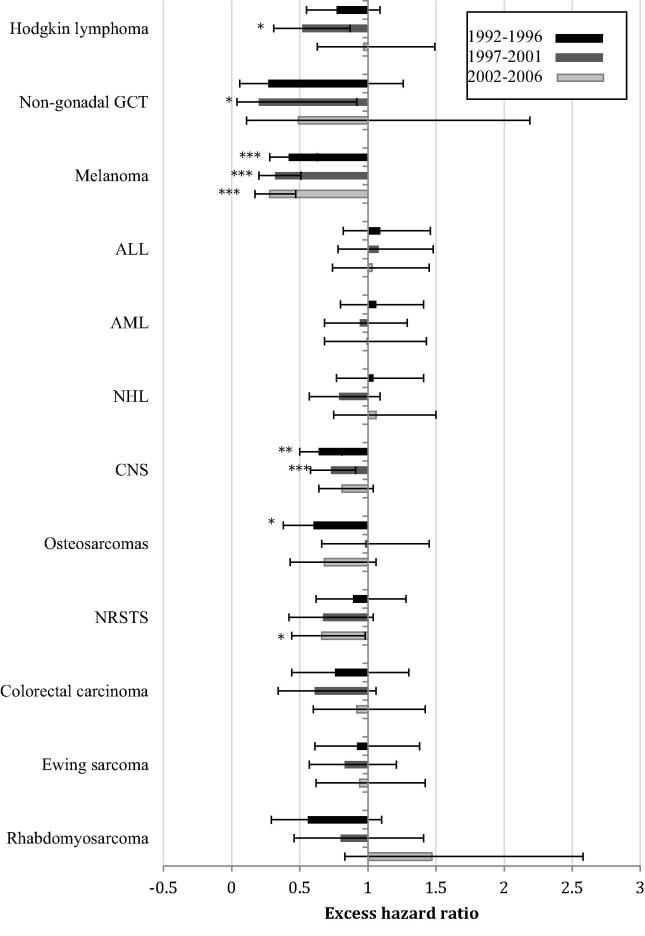
Excess hazard ratios for females compared with males for each time period and disease group. Error bars represent 95% confidence intervals. ALL, acute lymphoblastic leukaemia; AML, acute myeloid leukaemia; NHL, non-Hodgkin lymphoma; HL, Hodgkin lymphoma; GCT, germ cell tumour; CNS, central nervous system tumour; NRSTS, soft tissue sarcomas excluding rhabdomyosarcomas. Significance is indicated as ^*^P < 0.05, ^**^P < 0.01, ^***^P < 0.001.

Males had significantly worse survival than females during one or more of the earlier study periods for five cancer types: melanomas, CNS tumours (both *P* < 0.001), osteosarcomas, Hodgkin lymphomas and non-gonadal germ cell tumours (all *P* < 0.05). These differences had reduced to a non-significant level by 2002–2006 for all other than melanomas, largely due to improvements in the survival of male patients rather than deteriorations in the survival of female patients. During the most recent period, male patients with NRSTS also had significantly worse survival than females: the gender differences had not previously been significantly different (Fig. 3). There were no cancer types for which survival of male patients was better than survival of female patients.

### 3.4 Haematological malignancies

Survival for all major haematological malignancies increased significantly between 1992–1996 and 2002–2006. The annual average percentage increase for leukaemia was the highest among all TYA cancers at 3.2% for ALL and 3.9% for AML. Most improvement occurred between 1997–2001 and 2002–2006. Five-year survival also increased significantly between the earliest and latest periods for NHL and Hodgkin lymphoma.

### 3.5 CNS tumours

There were no significant changes in survival rates for either males or females with CNS tumours between 1992–1996 and 1997–2001. However, survival of male patients did increase significantly between 1997–2001 and 2002–2006, giving an overall improvement of 3.9% for all persons. Females maintained significantly better survival than males throughout the study period.

### 3.6 Bone tumours

Survival from Ewing sarcoma increased significantly between 1992–1996 and 2002–2006, most of the improvement being seen in males. Survival for osteosarcoma did not change significantly.

### 3.7 Soft tissue sarcomas

For NRSTS, survival improved significantly by 10.1%, the improvement limited to the period between 1992–1996 and 1997–2001. In contrast, survival from rhabdomyosarcoma did not significantly change, although there was a non-significant increase of 7.8% between the latter two study periods. There was an apparent reversal of gender differences in survival from rhabdomyosarcoma between the earliest and latest periods but the differences remained non-significant (Fig. 3).

### 3.8 Germ cell tumours

Survival for patients with testicular GCT increased significantly from 93.6% in 1992–1996 to 96.5% in 2002–2006. There were no significant changes in the survival of ovarian GCT or non-gonadal GCT.

### 3.9 Melanoma

Survival increased significantly by 5.0% for melanoma over the whole study period. Females maintained better survival than males throughout the entire period.

### 3.10 Carcinomas

Survival for breast carcinoma in females increased significantly by 2.4% per year and by 14.5% overall, mostly in the latter half of the study period. Survival for cervical carcinoma and colorectal carcinoma rose significantly by 8.4% and 10.4% respectively, predominantly in the first half of the study period. The improvement in colorectal carcinoma was more marked in males than females. There were no significant changes in survival of thyroid cancer or ovarian carcinoma.

### 3.11 International comparisons

Table 3 shows five-year survival rates from our study for 2002–2006 alongside reports for eight other populations in Europe, USA, Canada and Australia. One paper from the Netherlands [17] presented for males and females separately and the data were therefore not shown.

Five-year survival for the UK was similar to other populations for most types of cancer, although some results may warrant further investigation. The most recent cancer survival data for adolescents and young adults in the USA were published by Bleyer and colleagues [18]. These data were based on cancer registration data for 15–39 olds over the period 1985–2007. The results were generally similar to ours, with particularly large survival improvements in ALL and AML. UK data showed greater AAPC than US data for Ewing sarcoma (2.46 versus 0.75) and for breast carcinoma (2.37 versus 0.75). In contrast, five-year UK survival of osteosarcoma was 10–15% lower than for the USA [9,10] and for Germany [9]. Survival also appeared to be higher in Germany than in the UK for Hodgkin lymphoma and melanoma.

## 4 Discussion

Considerable improvements in five-year survival have been made for TYA patients in the UK since the early 1990s. In particular, we report here significant improvements in outcome for twelve of the eighteen types of cancer that make up the majority of TYA cancer in the UK. Improvements in survival were generally more marked between the latter two periods than the earlier two. We have not looked in this report at the factors that may underlie these observed improvements. However, the study periods we report here have encompassed several major changes in treatment that may have affected survival outcomes in TYA cancers. For instance, there has been a major shift in the treatment of acute lymphoblastic leukaemia in this age group from an ‘adult’ treatment strategy to a ‘paediatric’ treatment strategy following the publication of several reports of differential survival in adolescents treated according to paediatric strategies [19].

In addition, there have been several centrally driven initiatives that may have impacted on survival. The Calman-Hine report was published in 1995, at the end of the first period reported here, and made several key recommendations for the delivery of cancer diagnosis and care, with specific reference to the care of children and adolescents with cancer, and the role of specialised care for rare cancers [20]. The 1990s and 2000s saw the development of specialist TYA cancer centres in most major cities, affiliated with major cancer centres. Finally, the development of TYA multidisciplinary teams (MDTs) with both age and disease specialisation have played a central role in the management of many adolescent and young adult patients. The pivotal role of the TYA MDT was recognised in its inclusion as a core component in the delivery of cancer care in the national guidance for young people with cancer [11]. Nevertheless, areas of concern persist, particularly the apparent stasis in outcome figures for ovarian carcinoma, ovarian GCT, non-gonadal GCT, rhabdomyosarcoma and osteosarcoma. For some of the key malignancies characteristic of the adolescent and young adult years, bone sarcomas and alveolar rhabdomyosarcomas, overall survival remains around or under 50%. Moreover, there remains a survival gap between genders for some cancers.

The international data we discuss vary in terms of population sizes, age ranges, time periods, data collection methods, population coverage and survival analysis methods. The age range focus in particular is likely to influence apparent differences. Survival for ALL and to a lesser extent AML decreases with increasing age [6] which may partly explain the higher survival observed for ALL among 15–24 year olds in the UK compared with the 15–39 age group in the USA [18].

Survival for osteosarcoma in the USA [9,10] and Germany [9] was considerably higher than for the UK; in fact osteosarcoma had the largest percentage difference compared with the UK for any type of cancer in each of these three studies. Since the early 1980s much of North West Europe performed collaborative clinical trials in osteosarcoma under the auspices of the European Osteosarcoma Intergroup (EOI) or the Cooperative German-Austrian-Swiss Osteosarcoma Study Group (COSS). During the earlier time periods we report here, the chemotherapy regimens used by the EOI and COSS groups were different. Towards the end of the latter period both groups collaborated under the EURAMOS collaboration [21,22] and used the same chemotherapy protocols and it is of concern that TYA patients with osteosarcoma in the UK appear to have worse outcomes than those treated in Germany.

Differences in diagnostic inclusion criteria are likely to have contributed to some of the variation seen in the comparison with UK and international data. Non-malignant CNS tumours were included in our study but were excluded from several others (e.g. [5,18]), therefore the results are not directly comparable. UK cancer statistics generally include benign and borderline CNS tumours since, although not malignant, such tumours are responsible for a considerable number of deaths. Differences may also arise as a result temporal variations in the diagnostic classification systems used: for instance, pilocytic astrocytomas had a malignant behaviour code in ICD-O-2 but are now classified as non-malignant in ICD-O-3. Further comparative studies will be needed to accurately quantify international differences in survival, based on common protocols such as those undertaken by EUROCARE and CONCORD [23] but with a focus on specific tumour types. Future comparative studies would be strengthened by focusing on the relationships between service provision, including issues such as where patients are treated, clinical disease management and survival.

It is hoped that recent policy changes for TYA cancer services in the UK will impact on the outcomes, including survival outcomes, of young cancer patients. These improvements may be particularly marked in the rarer tumours considered to benefit most from age- or disease-specialist care. The data we report here set a baseline for future comparisons. We have confined our analysis to patients aged 15–24 in recognition that this age group is particularly vulnerable to falling in a gap between paediatric and adult health services, but acknowledge that selection of this age range may be more meaningful for some health systems and types of cancer than others.

## Conflict of interest statement

None declared.

## Figures and Tables

**Table 1:** 

Diagnosis	1992–1996						1997–2001						2002–2006					
	Persons		Males		Females		Persons		Males		Females		Persons		Males		Females	
	*n*	% cases	*n*	% cases	*n*	% cases	*n*	% cases	*n*	% cases	*n*	% cases	*n*	% cases	*n*	% cases	*n*	% cases
All cancers	8095	100	4262	100	3833	100	8183	100	4465	100	3718	100	9379	100	4954	100	4425	100
ALL	359	4	228	5	131	3	330	4	213	5	117	3	420	4	289	6	131	3
AML	344	4	188	4	156	4	286	3	146	3	140	4	282	3	150	3	132	3
NHL	543	7	341	8	202	5	595	7	372	8	223	6	633	7	389	8	244	6
HL	1352	17	688	16	664	17	1207	15	628	14	579	16	1358	14	693	14	665	15
CNS	1150	14	582	14	568	15	1144	14	598	13	546	15	1286	14	667	13	619	14
Osteosarcoma	200	2	126	3	74	2	213	3	133	3	80	2	206	2	136	3	70	2
Ewing sarcoma	149	2	87	2	62	2	215	3	127	3	88	2	186	2	122	2	64	1
NRSTS	269	3	134	3	135	4	262	3	142	3	120	3	297	3	156	3	141	3
Rhabdomyosarcoma	77	1	57	1	20	1	87	1	61	1	26	1	80	1	53	1	27	1
Testicular GCT	1000	12	1000	23			1176	14	1176	26			1243	13	1243	25		
Non-gonadal GCT	82	1	60	1	22	1	86	1	67	2	19	1	101	1	81	2	20	0
Melanoma	834	10	284	7	550	14	849	10	284	6	565	15	1094	12	354	7	740	17
Thyroid carcinoma	327	4	60	1	267	7	299	4	70	2	229	6	446	5	88	2	358	8
Breast carcinoma	113	1			113	3	113	1			113	3	99	1			99	2
Ovarian carcinoma	209	3			209	5	187	2			187	5	233	2			233	5
Cervical carcinoma	153	2			153	4	191	2			191	5	247	3			247	6
Ovarian GCT	92	1			92	2	93	1			93	3	120	1			120	3
Colorectal carcinoma	127	2	65	2	62	2	164	2	76	2	88	2	267	3	138	3	129	3
All other cancer diagnoses	715	8	362	8	353	9	686	8	372	8	314	8	781	8	395	8	386	9

ALL, acute lymphoblastic leukaemia; AML, acute myeloid leukaemia; NHL, non-Hodgkin lymphoma; HL, Hodgkin lymphoma; GCT, germ cell tumour; CNS, central nervous system tumour; NRSTS, soft tissue sarcomas excluding rhabdomyosarcoma, GCT, germ cell tumour.

**Table 2:** Five year relative survival estimates (%) with 95% confidence intervals (CI) for 15-2 year olds in the UK by period of diagnosis.

		Persons				Males				Females			
Diagnosis	Period	% Survival	95% CI		*P*-value	% survival	95% CI		*P*-value	% Survival	95% CI		*P*-value
All cancers	1992–1996	75.7	74.8	76.6		73.5	72.1	74.8		78.2	76.8	79.5	
1997–2001	78.6	77.7	79.5	<0.001	77.0	75.7	78.2	<0.001	80.6	79.3	81.8	0.005
2002–2006	82.2	81.4	82.9	<0.001	80.6	79.5	81.7	<0.001	83.9	82.8	85.0	<0.001

*5-year survival 80-100%*													
Hodgkin lymphoma	1992–1996	89.8	88.1	91.3		88.5	86.0	90.8		91.2	88.7	93.2	
1997–2001	94.1	92.6	95.3	<0.001	92.7	90.3	94.5	0.014	96.1	94.1	97.5	0.001
2002–2006	93.6	92.1	94.8	0.001	93.5	91.4	95.1	0.002	93.7	91.5	95.3	0.090
Ovarian GCT	1992–1996									94.9	87.8	98.1	
1997–2001									96.1	89.3	98.7	0.566
2002–2006									93.6	87.4	96.9	0.803
Testicular GCT	1992–1996					93.6	91.9	95.0					
1997–2001					95.6	94.2	96.6	0.040				
2002–2006					96.5	95.3	97.4	0.001				
Non-gonadal GCT	1992–1996	79.4	68.9	86.7		75.1	62.1	84.2		91.3	68.6	98.0	
1997–2001	74.6	63.9	82.5	0.606	70.3	57.7	79.7	0.668	89.8	64.3	97.7	0.907
2002–2006	81.3	72.2	87.7	0.708	79.1	68.5	86.5	0.466	90.3	65.8	97.7	0.476
Melanoma	1992–1996	88.7	86.3	90.7		82.2	77.2	86.2		92.0	89.4	94.1	
1997–2001	91.0	88.8	92.8	0.078	83.9	79.1	87.7	0.090	94.6	92.3	96.2	0.078
2002–2006	93.7	92.0	95.0	<0.001	88.0	84.1	91.0	<0.001	96.4	94.8	97.6	0.001
Thyroid carcinoma	1992–1996	100.0	100.0	100.0		100.0	100.0	100.0		100.0	100.0	100.0	
1997–2001	99.7	97.7	100.0	0.996	97.3	89.2	99.4	0.999	100.0	100.0	100.0	0.999
2002–2006	99.0	97.3	99.7	0.996	96.7	89.9	99.0	0.999	99.5	97.8	100.0	0.999
Breast carcinoma	1992–1996									67.6	58.0	75.4	
1997–2001									70.2	60.8	77.8	0.706
2002–2006									82.1	73.0	88.5	0.015
Cervical carcinoma	1992–1996									79.4	72.1	85.1	
1997–2001									87.3	81.6	91.3	0.046
2002–2006									87.8	82.9	91.3	0.027
*5-year survival 50–80%*													
ALL	1992–1996	44.7	39.5	49.7		45.2	38.7	51.6		43.7	35.0	52.0	
1997–2001	50.7	45.2	56.0	0.148	51.7	44.8	58.2	0.149	48.9	39.5	57.6	0.351
2002–2006	62.0	57.2	66.5	<0.001	62.4	56.5	67.7	<0.001	61.2	52.3	69.0	0.002
AML	1992–1996	43.7	38.4	48.9		43.7	36.5	50.6		43.8	35.9	51.4	
1997–2001	48.7	42.8	54.4	0.070	47.3	39.0	55.2	0.070	50.2	41.6	58.2	0.111
2002–2006	60.4	54.4	65.9	<0.001	60.1	51.8	67.4	<0.001	60.8	51.9	68.6	0.001
NHL	1992–1996	66.3	62.1	70.1		67.0	61.7	71.7		65.1	58.1	71.3	
1997–2001	71.8	68.0	75.2	0.043	70.0	65.0	74.4	0.045	74.7	68.5	80.0	0.023
2002–2006	78.4	74.9	81.4	<0.001	79.0	74.6	82.8	<0.001	77.3	71.5	82.1	0.002
CNS	1992–1996	75.4	72.8	77.8		71.2	67.4	74.7		79.7	76.1	82.8	
1997–2001	73.3	70.6	75.7	0.249	70.0	66.1	73.5	0.289	76.9	73.1	80.2	0.215
2002–2006	79.3	77.0	81.5	0.015	77.6	74.2	80.6	0.011	81.2	77.9	84.1	0.538
Osteosarcoma	1992–1996	50.6	43.5	57.3		43.7	34.9	52.1		62.4	50.3	72.4	
1997–2001	49.4	42.5	55.9	0.874	48.2	39.5	56.4	0.928	51.4	40.0	61.7	0.167
2002–2006	54.0	46.9	60.5	0.488	50.1	41.4	58.1	0.447	61.6	49.1	71.9	0.975
NRSTS	1992–1996	56.7	50.5	62.4		54.6	45.7	62.5		58.8	49.9	656.6	
1997–2001	68.5	62.5	73.8	0.003	63.5	55.0	70.8	0.122	74.4	65.6	81.4	0.005
2002–2006	66.8	61.1	71.9	0.009	61.6	53.5	68.8	0.210	72.6	64.4	79.2	0.010
Ovarian carcinoma	1992–1996									81.2	75.2	86.0	
1997–2001									76.8	70.0	82.3	0.249
2002–2006									79.7	73.9	84.4	0.78
Colorectal carcinoma	1992–1996	57.6	48.5	65.7		53.9	41.1	65.1		61.5	48.2	72.4	
1997–2001	68.5	60.8	75.0	0.052	61.9	50.0	71.8	0.059	74.2	63.6	82.1	0.119
2002–2006	68.0	62.0	73.2	0.023	66.8	58.2	74.0	0.020	69.2	60.5	76.5	0.265

*5-year survival <50%*													
Ewing sarcoma	1992–1996	33.0	25.6	40.6		29.9	20.7	39.7		37.2	25.4	49.1	
1997–2001	45.2	38.5	51.7	0.006	41.8	33.1	50.2	0.006	5.2	39.3	60.1	0.061
2002–2006	45.8	38.5	52.8	0.005	44.3	35.4	52.9	0.004	48.6	35.9	60.1	0.099
Rhabdomyosarcoma	1992–1996	27.3	17.9	37.6		24.6	14.4	36.3		35.1	15.7	55.4	
1997–2001	24.2	15.8	33.6	0.964	21.3	12.1	32.3	0.802	30.9	14.7	48.7	0.378
2002–2006	35.1	24.8	45.5	0.109	39.7	26.6	52.5	0.156	26.0	11.5	43.2	0.234

ALL, acute lymphoblastic leukameia; AML, acute myeloid leukaemia; NHL, non-Hodgkin lymphoma; GCT, germ cell tumour; CNS, central nervous system tumour; NRSTS, non-rhabdomyosarcoma soft tissue sarcomas, CI, confidence interval. *P*-value for excess hazard ratios comparing period 2 (1997–2001) with period 1 (1992–1996) and period 3 (2002–2006) with period 1.

**Table 3:** Published five year survival estimates (%) with 95% confidence intervals (in parentheses) for teenagers and young adults.

Publication	Country (diagnosis period and population coverage §)	Age range	ALL	AML	NHL	Hodgkin lymphoma	CNS	Osteosarcoma	Ewing sarcoma	Rhabdomyosarcoma	Gonadal GCT	Non-gonadal GCT	Melanoma	Breast (female)	Cervix	Colorectal
This publication	UK (2002–2006; fp)	15–24	62.0 (57–2-66.5)	60.4 (54.4–65.9)	78.4 (74.9–81.4)	93.6 (92.1–94.8)	79.3 (77.0–81.5)	54.0 (46.9–60.5)	45.8 (38.5–52.8)	35.1 (24.8–45.5)	96.3 (95.1–97.2)	81.3 (72.2–87.7)	93.7 (92.0–95.0)	82.1 (73.0–88.5)	87.8 (82.9–91.3)	68.0 (62.0–73.2)
15–19	64.7 (58.9–69.9)	61.5 (52.5–69.4)	81.5 (76.3–85.6)	93.1 (90.7–94.9)	81.0 (77.6–83.9)	61.0 (52.3–68.5)	44.9 (35.3–54.1)	36.8 (23.6–50.0)	94.5 (91.7–96.4)	86.7 (73.9–93.5)	92.6 (88.8–95.1)	78.0 (36.6–94.2)	66.9 (28.3–88.1)	84.9 (72.9–91.9)
20–24	56.9 (48.0–64.9)	59.5 (51.3–66.8)	76.0 (71.2–80.1)	94.0 (92.0–95.5)	78.4 (75.1–81.3)	39.8 (28.2–51.2)	46.9 (35.6–57.5)	32.3 (17.0–48.7)	97.0 (95.7–97.9)	75.7 (61.0–85.4)	94.1 (92.2–95.6)	82.9 (73.5–89.3)	80.7 (74.8–85.3)	63.6 (56.7–69.8)
Gondos et al. (2013)	USA (2002–2006; pp)	15–29	50.9 (46.0–55.8)	46.7 (41.8–51.6)	78.2 (75.7–80.7)	93.1 (91.7–94.5)		64.7 (58.8–70.6)	50.7 (43.3–58.1)		96.0 (95.0–97.0)	77.1 (69.8–84.4)	96.0 (95.0–97.0)	80.2 (76.9–83.5)	86.9 (84.2–89.6)	63.7 (59.0–68.4)
Germany (2002–2006; pp)	15–29	57.2 (50.5–63.9)	58.1 (50.5–65.7)	84.5 (81.0–88.0)	97.9 (96.9–98.9)		69.1 (61.1–77.1)	49.7 (40.1–59.3)		97.2 (96.2–98.2)	79.9 (70.3–89.5)	96.7 (95.5–97.9)	81.5 (76.8–86.2)	90.4 (86.7–94.1)	68.9 (61.5–76.3)
AIRTUM (2013)	Italy (2003–2008; pp)	15–19	74	77	88	95	81	59	28		100		95			
Desandes et al. (2013)	France (2000–2004; pp)	15–24	62.8 (50.0–73.2)	56.8 (39.4–70.8)	78.9 (67.4–86.7)	96.9 (93.6–98.5)	67.3 (57.1–75.7)	38.7 (22.0–55.1)	56.5 (34.3–73.8)	35.7 (13.0–59.4)	92.8 (88.7–96.9)		92.7 (85.3–96.5)			
Pole et al. (2013)	Canada (2004–2009; pp)	15–19	76 (65–89)													
20–29	69 (56–83)													
Carreira et al. (2012)	Portugal (1997–2006; pp)	15–19	50.0 (27.1–69.2) ≈	46.1 (19.2–69.6) acute non-lymphoblastic	67.9 (47.3–81.8)(excludes Burkitt)	96.7 (90.1–98.9)	56.4 (38.3–71.0) n/a	70.0 (45.1–85.3)	60.0 (31.8–79.7) includes related sarcomas	27.3 (0.07–53.9)	94.1 (78.5–98.5)	62.5 (22.9–86.1)	91.7 (53.9–98.9)	insuff cases	insuff cases	50.0 (15.2–77.5)
20–24	50.0 (22.9–72.2) ≈	44.4 (21.6–65.1)	61.2 (44.1–74.5)	94.8 (88.7–97.6)	83.1 (71.5–90.3) n/a	75.0 (40.8–91.2)	28.6 (4.1–61.2)	Insuff cases	94.7 (87.7–97.8)	54.6 (22.9–78.0)	83.3 (61.5–93.4)	68.0 (42.1–84.2)	92.3 (56.6–98.9)	76.5(48.8–90.5)
Bleyer (2011)	USA (2000–2007; pp)	15–39	49.1 (45.2–53.0)	49.4 (44.1–54.7)	74.6 (72.3–77.0)	92.2 (90.6–93.8)	64.6 (61.3–67.9) n/a	65.8 (57.4–74.2)	51.4 (40.6–62.2)	38.1 (26.3–49.9)			94.1 (93.1–95.1)	82.5 (81.3–83.7)	82.0 (80.0–84.0)	65.9 (63.2–68.6)
Pinkerton et al. (2010)	Australia (2000–2004; fp)	15–19	73.6 (62.5–81.8)	74.2 (61.3–83.3)	83.3 (75.3–88.9)	97.5 (94.0–98.9)										
20–29	47.1 (34.5–58.6)	62.5 (54.1–69.8)	81.1 (76.6–84.8)	95.1 (92.5–96.8)										
Gatta et al. (2009)	Europe (2000–2002)	15–24	49.5 (42.5–56.5)	59.1 (50.3–67.9)	74.4 (69.2–79.5)	93.1 (91.4–94.9)	61.7 (56.5–67.0) n/a	59.8 (51.2–68.5)	48.0 (35.3–60.6)				92.2 (89.6–94.9)	85.5 (79.2–91.8)	85.7 (73.2–98.1)	
UK and Ireland	52.6 (47.3–57.8)	49.1 (43.6–54.7)	69.6 (65.0–74.2)	92.9 (91.4–94.3)	57.2 (52.9–61.4) n/a	54.9 (47.3–62.5)	41.8 (33.1–50.5)				91.3 (89.3–93.2)	67.4 (58.7–76.1)	82.0 (76.5–87.5)	
Central Europe	50.1 (42.4–57.8)	47.4 (37.4–57.8)	69.4 (63.1–75.8)	94.7 (92.8–96.7)	62.1 (55.5–68.8) n/a	66.7 (56.8–76.6)	47.0 (33.0–61.0)				93.4 (90.0–95.9)	80.6 (69.6–91.6)	93.6 (87.5–99.8)	
Southern Europe	51.7 (43.5–59.9)	47.8 (38.8–56.8)	73.4 (68.5–78.3)	93.5 (91.6–95.4)	57.0 (50.7–63.3) n/a	60.1 (49.6–70.6)	33.6 (22.0–45.2)				92.8 (89.9–95.7)	75.8 (64.9–86.7)	81.0 (63.9–98.1)	
Eastern Europe	60.4 (38.6–82.2)	45.6 (17.6–73.5)	71.8 (57.4–86.2)	93.4 (89.2–97.7)	64.2 (51.0–77.5) n/a	52.0 (25.5–78.5)	50.0 (18.4–81.6)				86.3 (74.9–97.7)		83.3 (61.8–100)	
Northern Europe	59.5 (51.9–67.1)	47.0 (37.0–57.0)	74.8 (68.6–81.0)	95.4 (93.6–97.1)	65.5 (59.9–71.1) n/a	63.2 (52.3–74.1)	58.0 (40.8–75.2)				97.2 (95.6–98.8)	78.2 (62.4–94.1)	91.2 (84.9–97.6)	

ALL, acute lymphoblastic leukaemia; AML, acute myeloid leukaemia; NHL, non-Hodgkin lymphoma; GCT, germ cell tumour; CNS, central nervous system tumour. Survival rates with 95% confidence intervals (CI) that do not overlap with those for the UK are indicated in bold type. 95% CIs not included in the original publication have been calculated from the published standard error (SE) where available. CNS tumour rates for the UK include benign and borderline tumours. Where CNS tumour rates for other countries include only malignant tumours the comparison is indicated as n/a. §: Population coverage is indicated for each country as fp (full population) or pp (partial population).
